# Watching tetrahedral intercalation in transition metal hydroxides *in situ*

**DOI:** 10.1093/nsr/nwaf051

**Published:** 2025-02-18

**Authors:** Luis M Liz-Marzán

**Affiliations:** CIC biomaGUNE, Basque Research and Technology Alliance (BRTA), Spain; Ikerbasque, Basque Foundation for Science, Spain; CINBIO, Universidade de Vigo, Spain

Transition metal hydroxides (TMHs) can be used as model systems to understand nucleation and crystallization in hydroxide-rich minerals [1]. Beyond their fundamental significance, TMHs additionally find application in various fields, such as catalysis, energy storage, and electronic devices [2,3]. The synthesis of TMHs typically relies on wet chemistry, using OH^−^ concentration to drive the transformation of water/anion-coordinated metal ions [4]. Ultimately, a complex network of anion-coordinated metal polyhedra is obtained, containing metal ions with unconventional coordination numbers (UCN) together with the conventional octahedral (6-coordinated) structures. The way in which such polyhedra with UCN intercalate and deintercalate in the network plays a major role in the nucleation, growth, final composition, and the properties/functionality of the obtained TMHs [5]. However, the relevant dynamic behavior of polyhedra with UCN during TMH formation remains poorly understood due to the limitations of conventional *ex situ* characterization techniques.

**Figure 1.**
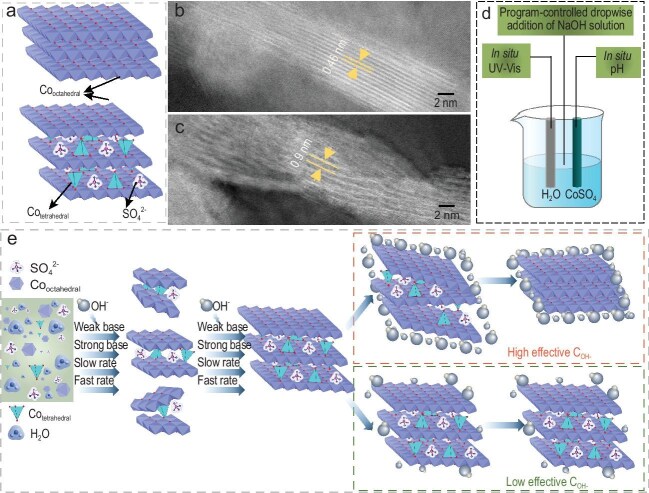
(a) Schematic view of the structures of Co(OH)_2_ with exclusive octahedral Co sites (top) and with both 4-coordinated tetrahedral and 6-coordinated octahedral Co sites (bottom). (b and c) Transmission electron micrographs showing the different interplanar spacing for Co(OH)_2_ with only 6-coordination (b) or 6/4-coordination (c) environments. (d) Schematic representation of the setup used for investigating the intercalation/deintercalation mechanism of tetrahedral Co^2+^ in Co(OH)_2_. (e) Illustration of the proposed intercalation/deintercalation mechanism.

An international collaboration team led by Prof. Minghua Huang from Ocean University of China, Dr. Saskia Heumann from the Max Planck Institute for Chemical Energy Conversion, Prof. Heqing Jiang from the Chinese Academy of Sciences, and Prof. Helmut Cölfen from the University of Konstanz, has now been able to confirm the presence of UCN in Co(OH)_2_ and how it affects the overall structure (Fig. 1a–c) [6]. Comprehension of the intercalation/deintercalation mechanism in tetrahedral Co^2+^ in Co(OH)_2_ was achieved through a multimodal *in situ* characterization suite. By simultaneous *in situ* pH determination (through a H^+^-selective electrode) and UV-Vis spectroscopy, both OH^−^ concentration and the evolving coordination environment of Co^2+^ were monitored in real time. It should be noted that specific UV-Vis signals for tetrahedral Co^2+^ facilitated the observation of its incorporation and release during TMH precipitation [7]. Another advantage of this system is the possibility to modulate the reaction rate by controlled introduction of NaOH and NH_3_ (Fig. 1d), thereby revealing further insights into the factors governing intercalation/deintercalation of undercoordinated polyhedra (tetrahedral Co^2+^). This combined approach uncovered a critical correlation between tetrahedral Co^2+^ intercalation and OH^−^ concentration. As shown in Fig. 1e, at early stages of Co(OH)_2_ formation, tetrahedral Co^2+^ is preferentially incorporated into the lattice, whereas its retention is largely dictated by the effective OH^−^ concentration.

This combined methodology significantly advances our fundamental understanding of TMH crystallization. Additionally, it provides a framework for the rational synthesis design of tunable coordination environments, which can be used to optimize TMH materials for applications such as oxygen evolution reaction (OER) catalysis. The unique combination of *in situ* techniques employed in this study represents a powerful tool to investigate other hydroxide-based materials beyond Co(OH)_2_ and subsequently design synthesis strategies toward enhanced material functionalities.

In summary, this work represents a significant step toward decoding the intercalation dynamics of polyhedra with UCN in TMHs. By bridging the gap between synthesis conditions and structural evolution, it offers a new paradigm for the controlled modulation of metal coordination environments. This approach holds great potential for advancing both fundamental research and the development of high-performance hydroxide materials for energy and environmental applications.
